# Alternative splicing of KRAS exon 4 promotes tumor progression via enhanced KRAS4A oncogenic activity

**DOI:** 10.1080/19768354.2025.2598971

**Published:** 2025-12-05

**Authors:** Namjoon Cho, Eunhye Kwon, Si-Eon Kim, Kee K. Kim

**Affiliations:** Department of Biochemistry, College of Natural Sciences, Chungnam National University, Daejeon, Republic of Korea

**Keywords:** KRAS, alternative splicing, oncogene, splicing factors, cancer progression

## Abstract

KRAS is a well-established oncogene that exhibits high-frequency mutations at cancer-driver hotspot loci across human cancers. While the oncogenic roles of mutant KRAS have been extensively investigated, the functional significance of KRAS splicing isoforms generated by exon 4 (E4) alternative splicing remains poorly understood. Here, we analyzed the expression patterns of *KRAS* E4 splicing variants in cancer tissues using The Cancer Genome Atlas (TCGA) data and found that certain cancer types exhibit relatively higher expression of the E4-included *KRAS* (*KRAS4A*) splicing variant compared to the E4-excluded KRAS (*KRAS4B*) splicing variant. Functional analysis revealed that the oncogenic properties of KRAS4A were significantly enhanced compared to those of KRAS4B. Furthermore, we identified RBM47 and PTBP1 as key regulators that promote *KRAS* E4 inclusion. Our findings demonstrate that RBM47- and PTBP1-mediated alternative splicing of *KRAS* contributes to enhanced tumor progression, highlighting *KRAS* alternative splicing as a promising therapeutic target for cancer treatment.

## Introduction

Alternative pre-mRNA splicing is a fundamental mechanism that generates multiple mRNA variants by selectively including or excluding exons from the pre-mRNA of a single gene. More than 95% of human multi-exon genes undergo alternative splicing, thereby creating transcriptome and proteome diversity (Pan et al. 2008; Bludau and Aebersold 2020). Splicing factors, which are differentially expressed in a cell type-specific manner, influence global alternative splicing patterns and can determine cell fate decisions (S. Choi, N. Cho and K. K. Kim 2023; S. Choi, N. Cho, E. M. Kim, et al. 2023). We previously conducted a comprehensive analysis of alternative splicing changes in cancer tissues compared with adjacent normal tissues and identified significant alterations in alternative splicing events across cancer driver genes (Cho et al. 2024a). Notably, we demonstrated that alterations of PBRM1 alternative splicing promote cancer immune evasion. These findings suggest that cancer cells may acquire advantages from altered alternative splicing patterns, including enhanced proliferation, survival, migration, and immune evasion capabilities. However, the functional roles of alternative splicing in cancer development and progression remain incompletely understood.

*KRAS* is one of the most frequently mutated oncogenes in human cancers, with missense mutations typically occurring at hotspot codons 12, 13, and 61 (Lee et al. 2022). *KRAS* mutations are highly prevalent in pancreatic cancer (∼74.0%), colorectal cancer (∼43.5%), and non-small cell lung cancer (∼29.7%), acting as oncogenic drivers through activation of downstream signaling pathways, including the extracellular signal-regulated kinase (ERK) pathway (Scharpf et al. 2022; Klomp et al. 2024). Activation of the ERK cascade by mutant KRAS promotes tumor progression by enhancing cell proliferation, epithelial-to-mesenchymal transition, and resistance to apoptosis. While extensive research has focused on the oncogenic functions of mutant KRAS, the significance of wild-type KRAS alternative splicing patterns remains poorly characterized.

The *KRAS* gene generates two major splicing variants through alternative splicing of exon 4 (E4): *KRAS4A*, which includes E4, and *KRAS4B*, which excludes it. Both E4 and the downstream exon 5 (E5) contain stop codons, resulting in the mutually exclusive translation of either E4 or E5 into the C-terminal amino acid sequence of the KRAS protein. This C-terminal region constitutes the hypervariable region (HVR), which is critical for membrane localization and functional specificity of KRAS splicing isoforms (Nussinov et al. 2017). Importantly, both E4 and E5 retain highly conserved protein-coding sequences across vertebrates, suggesting that this mutually exclusive selection of KRAS C-terminal sequences via alternative splicing plays a pivotal role in regulating KRAS function and determining cellular outcomes. However, most previous studies investigating KRAS function have primarily focused on KRAS4B, largely due to its predominant expression in many cell types and its well-characterized role in RAS signaling pathways. In contrast, KRAS4A has received comparatively limited attention, and its functional relevance in cancer pathogenesis remains underexplored.

In this study, we conducted a comprehensive analysis of *KRAS* E4 alternative splicing patterns across multiple cancer types. We found that a subset of cancer tissues exhibits preferential expression of *KRAS4A* compared to *KRAS4B*. Furthermore, we demonstrated that KRAS4A displays enhanced oncogenic activity relative to KRAS4B. Additionally, we identified specific splicing factors that regulate *KRAS* E4 inclusion. Collectively, our findings highlight the critical role of alternative splicing in modulating wild-type KRAS function and its contribution to tumor progression.

## Materials and methods

### Cell culture

HeLa, HCT116, and SK-N-SH cell lines were obtained from the American Type Culture Collection (ATCC) and maintained in Dulbecco's modified Eagle's medium (DMEM; WELGENE, LM001-05) supplemented with 10% (v/v) fetal bovine serum (FBS; Gibco, 12483-020) and 1% (v/v) penicillin/streptomycin (Corning, 30-002-CI, Corning). Cells were cultured at 37°C in a humidified atmosphere containing 5% CO₂ and passaged when reaching 80-90% confluency using 0.25% trypsin-EDTA (Gibco, 25200-056).

### TCGA data analysis

Percent spliced in (PSI) index values for *KRAS* E4 alternative splicing events were obtained from the TCGA SpliceSeq database (https://bioinformatics.mdanderson.org/TCGASpliceSeq/index.jsp, Version 2) (Ryan et al. 2016). PSI values represent the ratio of exon inclusion transcripts to total transcripts and range from 0 (complete exclusion) to 1 (complete inclusion). Fragments per kilobase of transcript per million mapped reads (FPKM) values in TCGA cancer tissues and corresponding adjacent normal tissues were downloaded from UCSC Xena (https://xena.ucsc.edu/) (Goldman et al. 2020). Only samples with both PSI and FPKM data available were included in the analysis to ensure data consistency.

### Plasmids and transfection

Flag-tagged RBM47 and Myc-tagged PTBP1 were cloned into pCMV-Tag2B and pCS3 + MT vectors, respectively, as previously described (Kim et al. 2011; Kim et al. 2019). Myc-tagged KRAS splicing isoforms (KRAS4A and KRAS4B) were cloned into the pCS3 + MT vector using PCR-amplified inserts. The primers used were as follows: Forward primer for both KRAS splicing isoforms, 5′-GGC CAG ATC TAC ATG ACT GAA TAT AAA CTT GTG G-3′; Reverse primer for the KRAS4A, 5′-GGC CCT CGA GCA CCC AGA TTA CAT TAT AAT GC-3′; Reverse primer for the KRAS4B, 5′-GCG CCT CGA GTT ACC ACT TGT ACT AGT ATG C-3′. The *KRAS* minigene construct, containing E3, E4 and E5 with their flanking intronic regions, was cloned into pEGFP-C3 vector using PCR products. The primer pairs used were as follows: KRAS E3, 5′-GCG CCT CGA GAG AAC AGT AGA CAC AAA ACA GGC-3′ and 5′-GCG CAA GCT TAC CAA AGC CAA AAG CAG TAC C-3′; KRAS E4, 5′-GCG CAA GCT TGG TTC CAG TTT CTT GAC TCA CC-3′ and 5′-GCG CGT CGA CGA GAT GGC GAA CTT AGG CAG-3′; KRAS E5, 5′-GCG CGT CGA CGA TGA CTT AGG TTT GCC AAT GTG-3′ and 5′-GCG CGG ATC CAT TAC CAC TTG TAC TAG TAT GCC-3′. Restriction enzyme recognition sites are underlined. Transfection was performed using the jetPEI reagent (Polyplus, 101000053) according to the manufacturer's instructions.

### Immunofluorescence microscopy

Cells were seeded in 8-well chamber slides (2 × 10⁴ cells/well; Thermo Fisher Scientific, 154534) for 24 h, and then transfected with 125 ng of KRAS expression vectors for 36 h. Transfected cells were fixed with 4% paraformaldehyde for 15 min at 25°C, permeabilized with phosphate-buffered saline (PBS) supplemented with 0.5% Triton X-100 for 10 min at 25°C, and blocked with an IF blocking buffer (5% goat serum, 1% bovine serum albumin, and 0.05% Tween 20 in PBS) for 1 h at 25°C. Mouse anti-Myc antibody (Invitrogen, R950-25; 1:2,500) was diluted in the IF blocking buffer and incubated with the samples for 12 h at 4°C. After washing with PBS supplemented with 0.05% Tween 20 (PBST), goat anti-mouse IgG Alexa Fluor 488 (Thermo Fisher Scientific, A11008; 1:500) and 4′,6-diamidino-2-phenylindole (DAPI; Thermo Fisher Scientific, D1306; 1:1,000) were applied for 1 h at 25°C. Images were captured using an LSM 880 with Airyscan (ZEISS) microscope. Fluorescent signals were quantified using ZEN software.

### Immunoblot analysis

Whole-cell lysates were obtained using the M-PER Mammalian Protein Extraction Reagent (Thermo Fisher Scientific, 78501) supplemented with the protease inhibitor cocktail (Roche, 11697498001), and denatured using the Laemmli Sample Buffer (Bio-Rad, 1610737) containing 5% β-mercaptoethanol (Sigma-Aldrich, T9281). The lysates were separated by SDS-PAGE and transferred to the nitrocellulose membrane (Merck Millipore, HATF00010). Membranes were blocked with PBST supplemented with 5% skim milk (ROCKLAND, B501-0500) for 1 h at 25°C, and then incubated with primary antibodies diluted in the blocking buffer for 12 h at 4°C. The following primary antibodies were used: anti-Myc (Invitrogen, 46-0603; 1:5,000), anti-Flag (Sigma-Aldrich, F3165; 1:20,000), anti-RAS (Cell Signaling Technology, 91054; 1:1,000), anti-p-ERK (Cell Signaling Technology, 9101; 1:1,000), anti-ERK (Cell Signaling Technology, 9102; 1:1,000), and anti-GAPDH (Meridian Bioscience, H86045P; 1:2,000) antibodies. After washing the membranes with PBST, horseradish peroxidase-conjugated anti-mouse IgG (Cell Signaling Technology, 7076; 1:5,000) or anti-rabbit IgG (Cell Signaling Technology, 7074; 1:5,000) antibodies, diluted in the blocking buffer, were applied and incubated for 1 h at 25°C. Membranes were then washed with PBST, and protein bands were detected using ECL solutions (Thermo Fisher Scientific, 34577 and 34075) on a ChemiDoc Imaging System (Bio-Rad).

### Colony formation assay

HeLa cells were seeded in 6-well plates (2.5 × 10⁵ cells/well) for 24 h and then transfected with 500 ng of KRAS expression vectors for 12 h. The cells were seeded in 6-well plates at a density of 500 cells/well and incubated for 10 days. Colonies were fixed with 4% paraformaldehyde for 10 min at 25°C and stained with 0.5% crystal violet in 99.5% methanol for 15 min. Plates were rinsed with running water, and images were captured. Colony numbers and sizes were quantified using ImageJ software.

### Migration and wound healing assays

After transfection with 500 ng of KRAS expression vectors for 24 h, HeLa cells were trypsinized, and 2 × 10⁵ cells in 150 µl of medium were seeded into Transwell chambers (8 µm pore size; SPL, 37224) for 20 h. The cells were fixed with methanol for 20 min and stained with Giemsa solution. After washing the chamber with PBS, non-migrated cells on the upper surface of the membrane were removed using a cotton swab. Bright-field images were captured using an inverted microscope (Olympus), and the number of migrated cells was quantified.

For the wound healing assay, HeLa cells transfected with 500 ng of KRAS expression vectors were cultured in 6-well plates until full confluency was reached. Wounds were made by scratching the cell monolayer with a 200 µl micropipette tip. After washing the wells with PBS, the cells were incubated in growth medium. Twenty-four hours after incubation, bright-field images were captured using an inverted microscope (Olympus), and wound closure areas were quantified using ImageJ software.

### Reverse transcription polymerase chain reaction (RT–PCR)

HeLa cells were seeded in 6-well plates (2.5 × 10⁵ cells/well) and incubated for 24 h. Cells were transfected with 1 µg of Flag-RBM47 or Myc-PTBP1 expression vectors for 36 h to examine alternative splicing patterns of the endogenous *KRAS* mRNA. For the minigene assay, cells were co-transfected with 950 ng of splicing factor (Flag-RBM47 or Myc-PTBP1) expression vectors and 50 ng of the *KRAS* minigene construct for 36 h. Total RNA was extracted using the Hybrid-R™ RNA extraction kit (GeneAll, 305-101) according to the manufacturer's instructions and reverse transcribed into cDNA. Detailed methods are described in previously published studies (Jung et al. 2023; Cho et al. 2024b). RT–PCR was performed using SYBR Green PCR Master Mix (GENETBIO, Q-9200) on an AriaMx real-time system (Agilent Technologies). The primers used were as follows: endogenous KRAS E4 splicing pattern, 5′-CTC AGG ACT TAG CAA GAA GTT ATG-3′ and 5′-CAT AAT TAC ACA CTT TGT CTT TGA CT-3′; E4 splicing pattern of *KRAS* minigene transcripts, 5′-CAT GGT CCT GCT GGA GTT CGT G-3′ and 5′-CAT AAT TAC ACA CTT TGT CTT TGA CT-3′. To calculate the E4 inclusion ratio, PCR band intensities were quantified using ImageJ software and normalized to the base-pair length of each PCR product.

### Statistical analysis

One-way ANOVA was performed using GraphPad Prism software, and Pearson correlation coefficients with corresponding *p*-values were calculated using Microsoft Excel. Bar graphs are presented as mean ± standard deviation (SD). Differences with *p*-values less than 0.05 were considered statistically significant.

## Results

### Expression of KRAS splicing variants in cancer tissues

**Figure 1.**
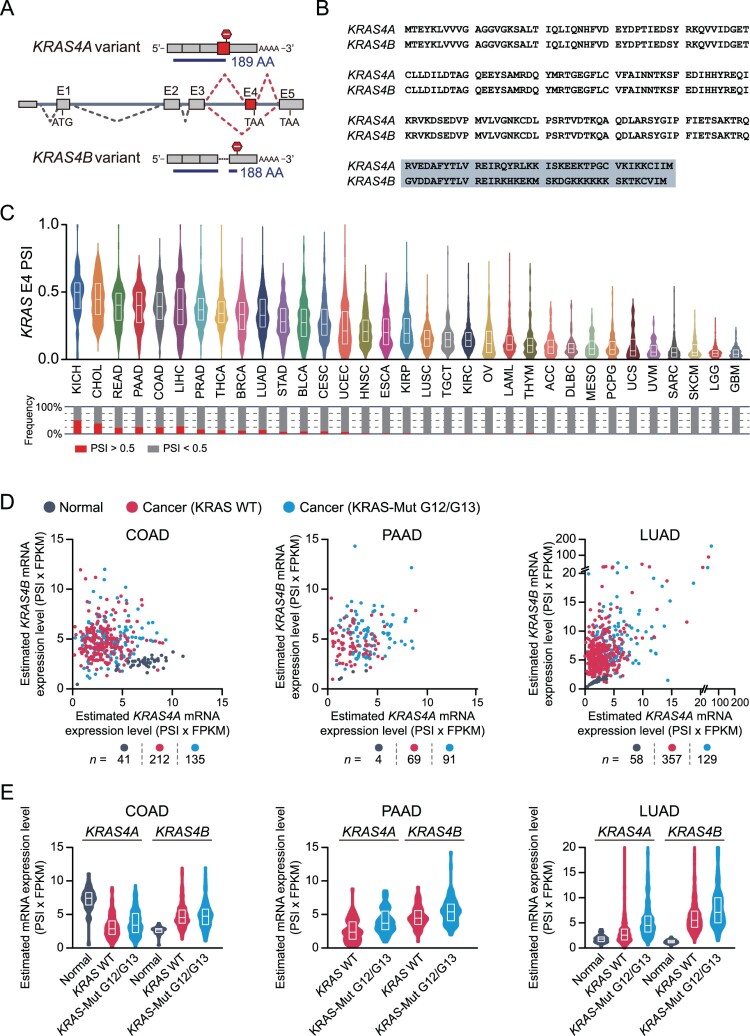
Expression of *KRAS4A* and *KRAS4B* splicing variants in cancer tissues across TCGA cancer types. (A) Schematic representation of human *KRAS* alternative splicing. The blue lines indicate protein-coding regions. E, exon; AA, amino acids. (B) Amino acid sequence alignments of KRAS4A and KRAS4B. The gray box represents the variable region generated by alternative splicing of E4. (C) Violin plots displaying PSI index values of *KRAS* E4 in cancer tissues across 33 TCGA cancer types. Boxes within violin plots indicate the median and quartiles. The bar graph at the bottom of the violin plots shows proportions of samples with PSI > 0.5. (D) Scatter plots showing TCGA tissues according to the estimated mRNA expression levels of *KRAS* splicing variants, with dots colored by *KRAS* somatic mutation status. (E) Violin plots of the estimated mRNA expression levels of *KRAS4A* and *KRAS4B* in TCGA tissues.

The human *KRAS* gene consists of one exon containing only the 5′ untranslated region and five exons encoding protein-coding sequences (Figure 1A). We numbered the protein-coding exons from E1 to E5, following the convention used in previous studies. Alternative splicing of the *KRAS* gene generates two major splicing variants, *KRAS4A* and *KRAS4B*, which differ in their inclusion or exclusion of E4. *KRAS4A* is the E4-included splicing variant, whereas *KRAS4B* is the E4-excluded splicing variant. Both E4 and E5 contain stop codons; thus, the inclusion or exclusion of E4 determines the site of translation termination, leading to distinct C-terminal amino acid sequences in the KRAS protein (Figure 1B).

We first analyzed the global alternative splicing patterns of *KRAS* E4 in cancer tissues. Based on percent spliced in (PSI) values of *KRAS* E4 from the TCGA SpliceSeq database, we performed a comprehensive analysis of E4 inclusion rates across 33 TCGA cancer types (Figure 1C) (Ryan et al. 2016). We found that *KRAS* E4 PSI values were broadly distributed across cancer tissues. Notably, in several cancer types, a subset of cancer tissues exhibited *KRAS* E4 PSI values greater than 0.5, suggesting that KRAS4A was more abundant than KRAS4B in certain cancer tissues.

Although PSI values of *KRAS* E4 quantify E4 inclusion rates from RNA-seq data, they do not capture the absolute mRNA expression levels of each *KRAS* splicing variant. To address this limitation, we estimated the mRNA expression levels of *KRAS4A* and *KRAS4B* splicing variants by multiplying the *KRAS* FPKM by the corresponding E4 PSI value (for *KRAS4A*) or by 1 – E4 PSI (for *KRAS4B*). In this analysis, we selected three cancer types with high prevalence of *KRAS* somatic mutations: colon adenocarcinoma (COAD), pancreatic adenocarcinoma (PAAD), and lung adenocarcinoma (LUAD). We classified patients with cancer by the presence or absence of hotspot mutations in the *KRAS* gene and estimated the mRNA expression patterns of *KRAS* splicing variants (Figure 1D, E). As shown in Figure 1D, scatter plots revealed variable estimated mRNA expression levels of KRAS4A and KRAS4B. We also found that the mRNA expression levels of KRAS4B were relatively higher than those of KRAS4A in COAD, PAAD, and LUAD cancer tissues. Overall, these results indicate that *KRAS4A* and *KRAS4B* splicing variants exhibit distinct expression patterns across cancer types.

### Activation of the ERK signaling pathway by overexpression of KRAS splicing isoforms

**Figure 2.**
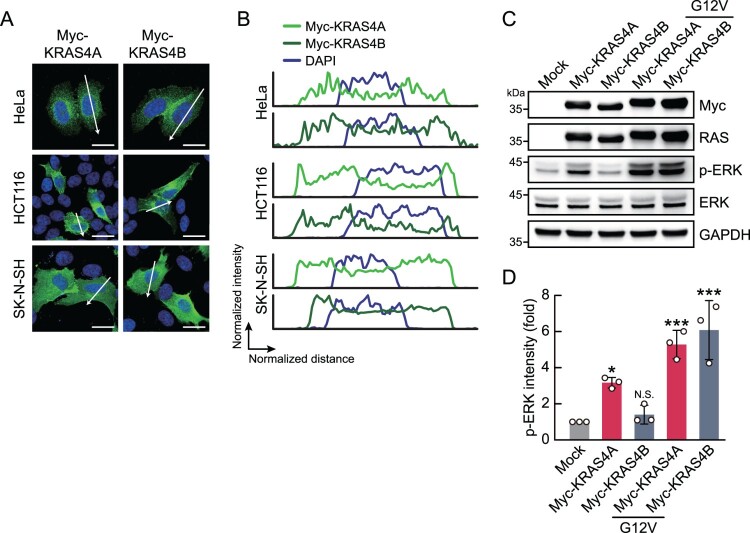
ERK phosphorylation activation by KRAS4A is greater than that by KRAS4B. (A) Immunofluorescence microscopy showing the subcellular localization of KRAS splicing isoforms. Myc-KRAS splicing isoform expression vectors were transfected into HeLa, HCT116, and SK-N-SH cells for 36 h, followed by immunofluorescence staining with an anti-Myc antibody (green). DAPI stained nuclei (blue). Scale bars, 20 µm. (B) Fluorescence intensities of Myc-KRAS splicing isoforms and DAPI along the arrow-indicated regions in (A). (C) Immunoblot analysis of ERK phosphorylation in HeLa cells 24 h after transfection with Myc-KRAS expression vectors. (D) Quantification of phosphorylated ERK (p-ERK) band intensities from immunoblot analysis. p-ERK signals were normalized to total ERK band intensities. Data are presented as mean ± SD. *p*-values were calculated using one-way ANOVA with Dunnett's multiple comparison test.

To examine the roles of KRAS splicing isoforms in cancer progression, we first analyzed subcellular localization patterns between KRAS splicing isoforms. Following overexpression of Myc-KRAS splicing isoforms in HeLa, HCT116, and SK-N-SH cells, we performed immunofluorescence analysis using an anti-Myc antibody (Figure 2A). While the subcellular localization patterns of KRAS splicing isoforms differed across cell lines, no isoform-specific differences were observed between KRAS4A and KRAS4B (Figure 2B). We next assessed their ability to activate the downstream ERK signaling pathway. We overexpressed Myc-KRAS4A or KRAS4B, with or without the G12D mutation, in HeLa cells under basal growth conditions for 24 h, and then analyzed ERK phosphorylation levels by immunoblot analysis (Figure 2C). This result showed that overexpression of Myc-KRAS4A enhanced ERK phosphorylation, whereas overexpression of Myc-KRAS4B had no significant effect (Figure 2D). Overexpression of the G12D mutant form of either Myc-KRAS4A or Myc-KRAS4B enhanced ERK phosphorylation, with no significant isoform-specific differences observed. These findings suggest that, in cancer tissues lacking *KRAS* hotspot mutations, increased KRAS4A expression via alternative splicing may contribute to activation of the ERK signaling pathway.

### Differential oncogenic activities between KRAS splicing isoforms

**Figure 3.**
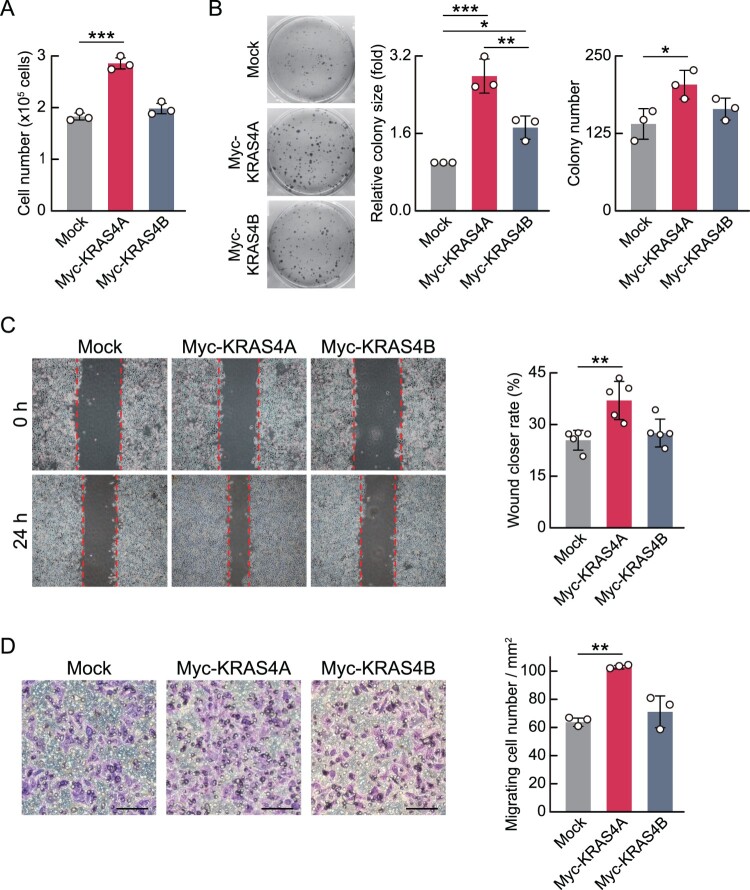
KRAS4A shows greater oncogenic properties than KRAS4B. (A) Bar graph showing the number of HeLa cells following overexpression of KRAS splicing isoforms. HeLa cells were transfected with Myc-KRAS expression vectors for 24 h and then seeded at equal numbers into culture wells. Three days after seeding, cell numbers were assessed by direct cell counting using a hemocytometer. Data are presented as mean ± SD. *p*-values were calculated using one-way ANOVA with Tukey's multiple comparison test. (B) Colony formation assay in HeLa cells after KRAS overexpression. Representative images are shown (left). Average colony number (middle) and colony size (right) are presented as bar graphs. Data are presented as mean ± SD. *p*-values were calculated using one-way ANOVA with Tukey's multiple comparison test. (C, D) Wound healing (C) and Transwell migration (D) assays in HeLa cells overexpressing KRAS splicing isoforms. Representative images were obtained under bright-field microscopy (left). Quantification of wound closure rates (C) and the number of migrating cells (D) are shown as bar graphs (right). Scale bar, 100 µm. Data are presented as mean ± SD. *p*-values were calculated using one-way ANOVA with Tukey's multiple comparison test.

Given the differences in ERK phosphorylation between KRAS splicing isoforms, we next analyzed their oncogenic properties. We first assessed the effects of KRAS splicing isoforms on cell proliferation and found that overexpression of Myc-KRAS4A promoted cell growth, whereas overexpression of Myc-KRAS4B showed no significant effect (Figure 3A). Moreover, a colony formation assay using HeLa cells following overexpression of KRAS splicing isoforms showed that KRAS4A exhibited greater cell proliferation-promoting activity compared to KRAS4B (Figure 3B).

We next analyzed the role of KRAS splicing isoforms in cancer cell metastasis. A wound healing assay showed that KRAS4A overexpression significantly increased wound closure rate, whereas KRAS4B overexpression did not (Figure 3C). In addition, a Transwell migration assay in HeLa cells overexpressing KRAS splicing isoforms showed that KRAS4A increased cell migration activity, while KRAS4B did not significantly affect cell migration (Figure 3D). Together, these findings suggest that KRAS4A not only enhances ERK phosphorylation more strongly but also exhibits greater oncogenic activity compared to KRAS4B.

### Splicing factors regulating alternative splicing of KRAS E4

**Figure 4.**
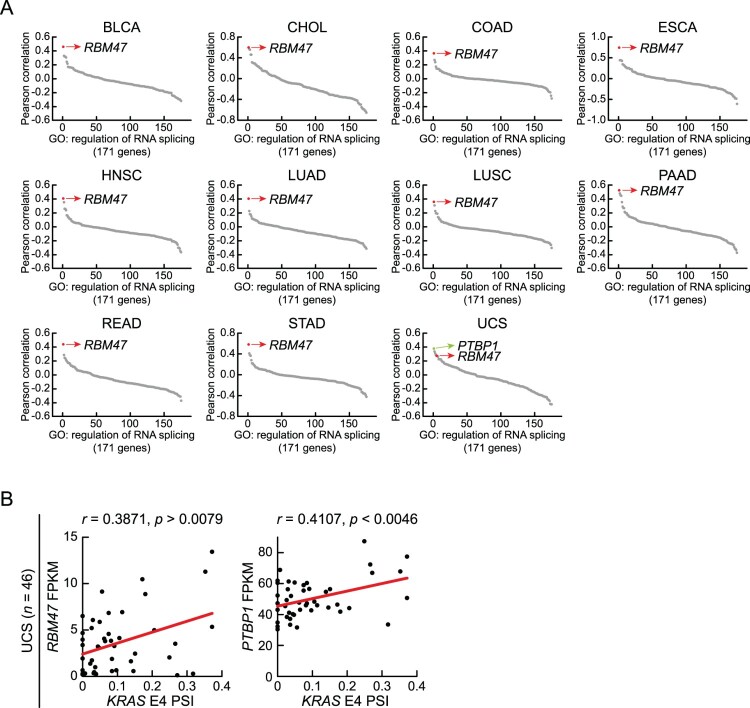
Pearson correlation analysis between mRNA expression levels of splicing factors and PSI values of *KRAS* E4. (A) Dot plots displaying splicing factors according to the Pearson correlation coefficient between FPKM values of splicing factors and the PSI values of *KRAS* E4 across individual cancer tissues within each TCGA cancer type. Colored dots indicate RBM47 (red) and PTBP1 (green). (B) Scatter plots of *KRAS* E4 PSI versus FPKM of *RBM47* (left) or *PTBP1* (right) in individual uterine carcinosarcoma (UCS) cancer tissues. Pearson correlation coefficients and *p*-values are presented.

To elucidate the regulatory mechanism underlying the alternative splicing of *KRAS* E4 in cancer tissues, we focused on the expression patterns of splicing factors. In our previous approach, we identified potential regulators of specific alternative splicing events by performing Pearson correlation analysis using mRNA expression levels within a defined splicing factor gene set (Choi et al. 2022; Cho et al. 2024a). Based on this strategy, we conducted a Pearson correlation analysis between PSI values of *KRAS* E4 and mRNA expression levels of splicing factors across TCGA cancer tissue datasets (Figure 4A). This result showed that the mRNA expression levels of *RBM47* exhibited the strongest positive correlation with PSI values of *KRAS* E4 across 10 cancer types. In uterine carcinosarcoma (UCS), PSI values of *KRAS* E4 showed the strongest positive correlation with *PTBP1* mRNA expression levels. Scatter plots of UCS cancer tissues confirmed positive correlations between PSI values of *KRAS* E4 and mRNA expression levels of *RBM47* and *PTBP1* (Figure 4B).

**Figure 5.**
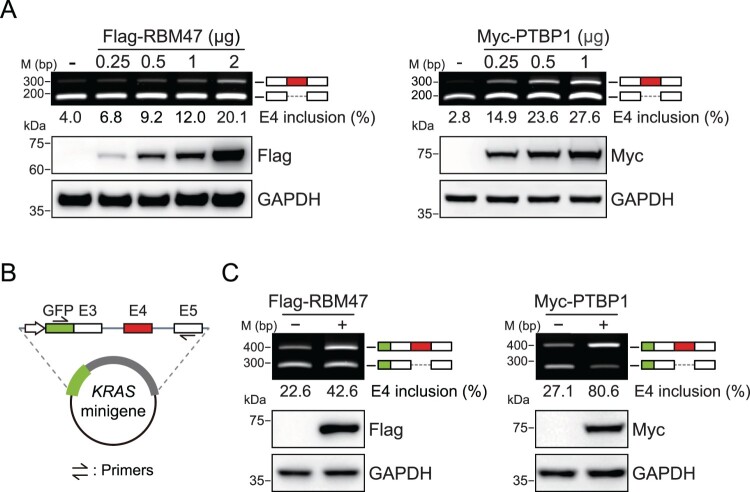
RBM47 and PTBP1 regulate alternative splicing of *KRAS* E4. (A) RT-PCR (top) and immunoblot (bottom) analyses showing E4 inclusion in *KRAS* pre-mRNA following overexpression of RBM47 (left) or PTBP1 (right) in HeLa cells for 36 h. (B) Schematic of the *KRAS* minigene construct. (C) RT-PCR (top) and immunoblot (bottom) analyses of alternative splicing patterns of *KRAS* minigene transcripts. HeLa cells were co-transfected with the *KRAS* minigene plasmid and RBM47 (left) or PTBP1 (right) expression vectors for 36 h.

Based on these profiles, we hypothesized that RBM47 and PTBP1 regulate the alternative splicing of *KRAS* E4, consistent with the positive correlations observed in TCGA cancer tissues. To investigate the effects of RBM47 and PTBP1 on *KRAS* E4 alternative splicing, we transfected HeLa cells with Flag-RBM47 or Myc-PTBP1 expression vectors and quantified E4 inclusion in *KRAS* mRNA by RT–PCR using a primer pair binding to the upstream (E3) and downstream (E5) exons, resulting in the amplification of the *KRAS4A* splicing variant as a higher-size DNA band and *KRAS4B* as a lower-size DNA band (Figure 5A). We found that RBM47 or PTBP1 overexpression induced E4 inclusion in *KRAS* mRNA. Next, we cloned the *KRAS* minigene construct containing E4, flanking E3 and E5, as well as *KRAS* introns (Figure 5B). Co-transfection of the *KRAS* minigene construct with Flag-RBM47 or Myc-PTBP1 expression vectors into HeLa cells confirmed that both RBM47 and PTBP1 induced E4 inclusion in *KRAS* minigene transcripts, supporting their role in regulating *KRAS* E4 alternative splicing through the recognition of E4 and its flanking RNA sequences (Figure 5C). Overall, our findings indicate that RBM47 and PTBP1 regulate the alternative splicing of *KRAS* E4 in human cancers, and that the resulting KRAS4A splicing isoforms may promote cancer cell proliferation and metastasis.

## Discussion

In this study, we revealed that E4 inclusion in *KRAS* mRNA through alternative splicing generates the KRAS4A splicing isoform with higher oncogenic properties than the KRAS4B splicing isoform, even in the absence of oncogenic hotspot mutations. Additionally, we identified that the mRNA expression of splicing factors RBM47 and PTBP1 positively correlates with *KRAS* E4 inclusion rates in cancer tissues and confirmed their roles in enhancing E4 inclusion in *KRAS* mRNA. These findings highlight a mechanism by which the oncogenic activity of wild-type KRAS depends on its E4 alternative splicing patterns.

Somatic mutations of the *KRAS* gene at hotspot codons are well-established drivers of cancer development (Cook et al. 2021). A recent study has shown that a subset of individuals without cancer contain cells with *KRAS* oncogenic mutations, potentially leading to tumor formation (Hill et al. 2023). Understanding the oncogenic activities of mutant KRAS is crucial for identifying mechanisms of cancer development and progression. However, most human cancers exhibit no mutations in *KRAS*, and this is also observed at measurable frequencies in COAD, LUAD, and PAAD. Therefore, we explored the possibility that RNA-level events, such as alternative splicing, could modify KRAS activity and contribute to tumor progression. Specifically, we demonstrated that KRAS4A possesses markedly higher oncogenic activity than KRAS4B by enhancing the ERK signaling pathway, cell proliferation, and metastatic potential. These results provide evidence that cells can acquire oncogenic phenotypes not only from somatic mutations in the *KRAS* gene but also from modulation of post-transcriptional regulation of KRAS through E4 inclusion.

Although the human *KRAS* gene produces two distinct alternative splicing isoforms, previous studies have primarily focused on KRAS4B when investigating the functional roles of KRAS. In our analysis, we also observed that HeLa cells expressed *KRAS4B* splicing variants at levels exceeding 95% (Figure 5A). Additionally, we examined *KRAS* E4 alternative splicing patterns across various cancer cell lines and found that nearly all of them predominantly express the *KRAS4B* splicing variant, in contrast to the broader distribution of E4 inclusion rates observed in the TCGA cancer tissue database analysis. We speculate that the in vitro culture conditions of cancer cell lines may promote strong E4 exclusion in *KRAS* mRNA. This exclusion pattern of *KRAS* E4 in cancer cell lines likely explains why most previous studies have mainly focused on the KRAS4B splicing isoform. These inherent limitations of cancer cell line – based models also restricted our ability to evaluate the oncogenic activities of endogenous KRAS4A, as the low abundance of *KRAS4A* transcripts in these cells was insufficient to reveal clear phenotypic changes upon depletion. To fully elucidate the physiological roles of KRAS4A, establishing tumor models that express KRAS with high E4 inclusion rates would be necessary. Subsequently reducing KRAS4A expression in such models could uncover the functional significance of KRAS4A and provide insights into its potential as a therapeutic target. Our analysis of TCGA datasets revealed that *KRAS4A* splicing variants are expressed at levels that could potentially influence tumor progression, with some cancer tissues even exhibiting *KRAS4*A as the dominant splicing variant over *KRAS4B*. A recent *KRAS* study also emphasized the importance of KRAS4A in tumors and found that it has differential roles compared to KRAS4B. KRAS4A and KRAS4B display notable structural differences, with KRAS4A exhibiting higher thermal stability than KRAS4B (Whitley et al. 2024). Moreover, KRAS4A has a greater impact on modulating energy metabolism by directly interacting with hexokinase 1 (HK1) compared to KRAS4B (Amendola et al. 2019). These findings, together with our cancer database profiling and molecular analyses, underscore the critical role of KRAS4A in cancer progression and highlight the need for further investigation.

Additionally, we found positive correlations between *KRAS* E4 inclusion rates and mRNA expression levels of RBM47 and PTBP1, and we further confirmed that these splicing factors regulate *KRAS* E4 alternative splicing. Another study has identified that the splicing factor RBM39 also regulates the alternative splicing of *KRAS* (Chen et al. 2021). Although KRAS4A has been recognized as a minor splicing isoform, the E4 sequence is evolutionarily conserved, and we found that it has distinct roles in signal transduction, cell proliferation, and metastatic potential compared to KRAS4B. These findings suggest that systematic regulation of *KRAS* E4 alternative splicing, through the expression and activation of various splicing factors, determines the expression pattern of KRAS4A and KRAS4B, thereby influencing cell fate. However, our study provides only preliminary evidence toward elucidating the regulatory mechanisms of *KRAS* E4 alternative splicing and its implications in the regulation of proliferation and differentiation. Another limitation of our study is that we did not investigate whether the oncogenic properties of KRAS4A affect human tumor development and progression; consequently, the clinical relevance of KRAS4A in patients with cancer remains unclear. Further studies should assess the significance of KRAS4A in tumor progression across different cancer types and evaluate its clinical implications, including responses to anti-cancer agents affecting the ERK signaling pathway (Singhal et al. 2024). We are also exploring the development of alternative splicing-modulating agents, such as antisense oligonucleotides targeting *KRAS* E4 inclusion, as a potential novel anti-cancer therapeutic strategy.

In summary, we analyzed the alternative splicing patterns of *KRAS* E4 across 33 cancer types and found that a subset of cancer tissues expressed *KRAS4A* splicing variants at higher levels than *KRAS4B* splicing variants. We demonstrated that the KRAS4A splicing isoform exhibited greater oncogenic activities than the KRAS4B splicing isoform. Finally, we identified RBM47 and PTBP1 as regulators of *KRAS* E4 alternative splicing in cancer. We believe that our findings broaden the current perspective on the oncogenic activity of KRAS by highlighting the role of alternative splicing and underscore its potential as a target for the development of cancer therapeutics.
